# Supplementary material for: Modality-specific tracking of attention and sensory statistics in the human electrophysiological spectral exponent
Source: eLife. 2021 Oct 21;10:e70068. doi: 10.7554/eLife.70068 (PMC8585481; doi:10.7554/eLife.70068)
Supplement: Supplementary file 1. [file elife-70068-supp1.docx]

| **Stimulus tracking model at electrode Cz** | | | | | |
| --- | --- | --- | --- | --- | --- |
|  | **EEG spectral exponent** | | | | |
| *Predictors* | *Estimates* | *std. Error* | *CI* | *t-value* | *p* |
| Intercept | 1.743 | 0.259 | 1.236 – 2.251 | 6.731 | **<0.001** |
| Auditory spectral exponent | 0.012 | 0.003 | 0.006 – 0.017 | 4.243 | **<0.001** |
| Attention | -0.013 | 0.006 | -0.025 – -0.001 | -2.172 | **0.0299** |
| Visual spectral exponent | -0.001 | 0.003 | -0.007 – 0.004 | -0.418 | 0.6758 |
| Trial number | 0.000 | 0.000 | 0.000 – 0.000 | 4.381 | **<0.001** |
| Resting state EEG exponent | -0.206 | 0.175 | -0.549 – 0.138 | -1.171 | 0.2415 |
| Auditory spectral exponent x Attention | -0.005 | 0.003 | -0.010 – 0.001 | -1.778 | 0.0754 |
| Visual spectral exponent x Attention | -0.003 | 0.003 | -0.008 – 0.003 | -0.948 | 0.3434 |
| **Random Effects** | | | | | |
| σ^2^ | 0.08 | | | | |
| τ_00_ _Sub_ | 0.04 | | | | |
| τ_11_ _Sub.Attention_ | 0.00 | | | | |
| ρ_01_ _Sub_ | 0.37 | | | | |
| N _Sub_ | 24 | | | | |
| Observations | 9940 | | | | |
| Marginal R^2^ / Conditional R^2^ | 0.020 / 0.332 | | | | |

**Supplementary file 1.** The table shows model coefficients (standardized betas), standard errors, confidence intervals, t-values, and p-values for the stimulus tracking model at electrode Cz.
