# Supplementary material for: Modality-specific tracking of attention and sensory statistics in the human electrophysiological spectral exponent
Source: eLife. 2021 Oct 21;10:e70068. doi: 10.7554/eLife.70068 (PMC8585481; doi:10.7554/eLife.70068)
Supplement: Supplementary file 2. [file elife-70068-supp2.docx]

| **Table S2: Stimulus tracking model electrode Oz** | | | | | |
| --- | --- | --- | --- | --- | --- |
|  | **EEG spectral exponent** | | | | |
| *Predictors* | *Estimates* | *std. Error* | *CI* | *t-value* | *p* |
| Intercept | 1.152 | 0.244 | 0.674 – 1.630 | 4.719 | **<0.001** |
| Auditory spectral exponent | 0.006 | 0.003 | -0.000 – 0.012 | 1.865 | 0.0622 |
| Attention | -0.040 | 0.009 | -0.058 – -0.023 | -4.479 | **<0.001** |
| Visual spectral exponent | 0.013 | 0.003 | 0.007 – 0.019 | 4.100 | **<0.001** |
| Trial number | 0.000 | 0.000 | 0.000 – 0.000 | 3.250 | **0.0012** |
| Resting state EEG exponent | 0.001 | 0.186 | -0.363 – 0.365 | 0.005 | 0.9960 |
| Auditory spectral exponent x Attention | 0.003 | 0.003 | -0.003 – 0.009 | 0.927 | 0.3538 |
| Visual spectral exponent x Attention | 0.003 | 0.003 | -0.003 – 0.009 | 1.015 | 0.3100 |
| **Random Effects** | | | | | |
| σ^2^ | 0.10 | | | | |
| τ_00_ _Sub_ | 0.09 | | | | |
| τ_11_ _Sub.Attention_ | 0.00 | | | | |
| ρ_01_ _Sub_ | 0.13 | | | | |
| N _Sub_ | 24 | | | | |
| Observations | 9940 | | | | |
| Marginal R^2^ / Conditional R^2^ | 0.010 / 0.497 | | | | |

**Supplementary file 2.** The table shows model coefficients (standardized betas), standard errors, confidence intervals, t-values, and p-values for the stimulus tracking model at electrode Oz.
